# Naringin alleviates methotrexate-induced liver injury in male albino rats and enhances its antitumor efficacy in HepG2 cells

**DOI:** 10.1042/BSR20193686

**Published:** 2020-06-10

**Authors:** Hany Elsawy, Abdulmohsen I. Algefare, Manal Alfwuaires, Mahmoud Khalil, Omar M. Elmenshawy, Azza Sedky, Ashraf M. Abdel-Moneim

**Affiliations:** 1 Department of Chemistry, Faculty of Science, King Faisal University, Al-Ahsa, Saudi Arabia; 2 Department of Chemistry, Faculty of Science, Tanta University, Tanta, Egypt; 3 Department of Biological Sciences, Faculty of Science, King Faisal University, Hofuf 31982, Al-Ahsa, Saudi Arabia; 4 Department of Biological Sciences, Faculty of Science, Beirut Arab University, Beirut, Lebanon; 5 Department of Zoology, Faculty of Science, Alexandria University, Alexandria, Egypt; 6 Department of Zoology, Faculty of Science, Al-Azhar University, Cairo, Egypt

**Keywords:** Bax/Bcl-2 ratio, HepG2 cells, liver oxidative damage, methotrexate, Naringin

## Abstract

Methotrexate (MTX) is an efficient chemotherapeutic and immunosuppressant drug, but the hepatotoxicity of MTX limits its clinical use. Naringin (Nar) is a flavonoid derived from *Citrus paradise*, and has been shown to possess several pharmacological activities, including free-radical scavenging and antioxidant properties. In the present study, we first tested the possible protective effects of multiple doses of Nar against MTX-induced acute hepatotoxicity in rats, and then we investigated the growth inhibition and apoptotic effects of MTX and/or Nar against the HepG2 hepatocarcinoma cell line. Our *in vivo* results showed that Nar significantly reduced MTX-induced increases in serum alanine aminotransferase, aspartate aminotransferase, alkaline phosphatase and total bilirubin levels. Nar also reduced MTX-induced oxidative stress by significantly reducing liver malondialdehyde (MDA) and nitric oxide (NO) content and increasing superoxide dismutase (SOD), catalase (CAT), glutathione peroxidase (GPx), glutathione reductase (GR), and glutathione (GSH). In addition, Nar significantly counteracted MTX-induced increases in hepatic interleukin-6 and tumor necrosis factor-α (TNF-α). Further, Nar greatly protected hepatocyte ultrastructure against MTX-induced injury. In contrast, *in vitro* MTX and/or Nar treatment of HepG2 cells for 48 h exhibited a cytotoxic effect and induced apoptosis in a dose-dependent manner mediated by a significant increase in the Bax/Bcl-2 protein expression ratio. Noticeably, Nar potentiated the MTX effect on the Bax/Bcl-2 ratio. In conclusion, Nar decreased MTX-induced functional and ultrastructural liver damage in a tumor-free animal model. Also, our data introduce MTX and Nar as promising antiproliferative agents with a distinctive mode of action, inducing apoptosis in HepG2 tumor cells through activation of Bax and down-regulation of Bcl-2 protein expression.

## Introduction

Methotrexate (MTX), a folic acid antagonist, is widely used in the treatment and prophylaxis of several types of malignant tumors [1,2]. However, MTX is known to induce liver damage in animals, and more than 10% of patients receiving MTX [3]. Several studies have shown that MTX toxicity is mediated by oxidative stress and dysregulation of antioxidant defenses, thereby increasing the generation of reactive oxygen species (ROS) as superoxide anions, hydrogen peroxide and hydroxyl radicals [4]. ROS formation can initiate lipid peroxidation (LPO) and the release of inflammatory mediators, including tumor necrosis factor-α (TNF-α) and inducible nitric oxide synthase (iNOS) [5]. TNF-α is a key factor in liver and kidney homeostasis. TNF-α activates proapoptotic (mainly caspases) and anti-apoptotic (mainly NF-κB pathways) [6,7]. Increased expression of TNF-α has been reported in a model of MTX-induced hepatic, renal and intestinal damage [8]. The proinflammatory effect of TNF-α is mediated through NF-κB-regulated proteins, such as iNOS and cyclooxygenase-2. [9]. Nitric oxide (NO) is a free radical molecule with a multitude of physiological functions. This highly reactive molecule is synthesized from l-arginine by a group of isoenzymes collectively termed as NO synthases (NOSs). The role of iNOS has been implicated in the pathogenesis of MTX-induced toxicity [10]. Therefore, attenuation of oxidative stress can represent an effective strategy to protect against MTX-induced hepatotoxicity.

Recently, several reports have highlighted the role of antioxidants in preventing diseases through free radical scavenging mechanisms [11]. Naringin (4′,5,7-trihydroxyflavanone-7-rhamnoglucoside) (Nar) is a bioflavonoid and polyphenolic compound that is found in grapefruit, orange and cooked tomato paste and is associated with citrus herb species [12]. Studies have shown that Nar has a broad spectrum of biological and pharmacological activities, including hepatoprotective [13], anticarcinogenic [14], antidiabetic [15] and anti-inflammatory effects [16].

According to the latest world cancer statistics published by the International Agency for Research on Cancer (IARC)-Global Cancer Observatory (GCO) (Globocan 2018), liver cancer is ranked fourth on the list of cancer-related deaths. Based on IARC statistics, the estimated number of deaths from liver cancer in 2018 is approximately 0.8 million, and the mortality burden projections worldwide will be increased by 66% in 2040 [17]. Few reports have highlighted the effects of Nar in HepG2 cells, including restraining inflammatory markers such as C-reactive protein and TNF-α, quenching NF-κB and ERK1/2 activities [18], and inducing apoptosis via the mitochondrial pathway [19], and via the up-regulation of miR-19b and Bax/Bcl-2 protein ratio expression [20]. In addition, it has been reported that the antihyperglycemic effects of Nar in HepG2 cells are associated with the up-regulation of phospho-AMP-activated protein kinase (pAMPK), a key enzyme in the regulation of cellular energy homeostasis [21,22]. In a very recent study, the polymeric micellization of Nar with pluronic F-68, a nonionic surfactant, was shown to enhance the antitumor activity of Nar against HepG2, MCF-7 and Caco-2 cells. Furthermore, Nar–PF68 micelles inhibit tumor growth in Ehrlich ascites carcinoma-bearing mice [23]. To the best of our knowledge, the dual effect of Nar and MTX on hepatocellular carcinoma (HCC) has not been investigated. Considering the abovementioned data, first, we aimed to evaluate the protective potential of Nar on MTX-induced hepatotoxicity in a rat model. Second, we aimed to uncover the effect of the cotreatment of Nar and MTX on the HepG2 HCC cell line.

## Materials and methods

### Animals and treatments

The animal work was conducted at King Faisal University (Al-Ahsa, Saudi Arabia). Animal care and experimental procedures were approved and licensed by the research ethics committee at King Faisal University (reference number: KFU-REC/2019-03-05). Male albino rats were obtained from an animal facility at the Faculty of Science, King Saud University, Saudi Arabia. Rats (220 ± 30 g) were housed in stainless steel wire-bottomed cages and placed in a well-ventilated animal house, maintained for 2 weeks for to acclimatize with food and water *ad libitum*, and subjected to a natural 12-h light:dark cycle. Rats were divided randomly into five groups (*n*=6 rats each) as follows:

- Control group: Rats received daily intraperitoneal (ip) administration of saline as a vehicle.
- MTX group: Rats received a single ip dose of MTX (20 mg/kg) on the fourth day of the experiment.
- MTX+ Nar20 group: Rats received a daily ip dose of Nar (20 mg/kg) and a single ip dose of MTX (20 mg/kg) on the fourth day of the experiment.
- MTX+ Nar40 group: Rats received a daily ip dose of Nar (40 mg/kg) and a single ip dose of MTX (20 mg/kg) on the fourth day of the experiment.
- MTX+ Nar80 group: Rats received a daily ip dose of Nar (80 mg/kg) and a single ip dose of MTX (20 mg/kg) on the fourth day of the experiment.

Nar (Cat. No. 71162) and *MTX* (Cat. No. M9929) have been purchased from Sigma–Aldrich, U.S.A. The drugs were dissolved in DMSO/saline with the final concentration of 1% DMSO. The duration of the experiment was 10 days. MTX and Nar doses were selected according to previous reports [5,24,25].

### *In vivo* sample collection and biochemical assays

At the end of the experimental period, rats were anesthetized with diethyl ether and killed by cervical dislocation. Blood samples were collected immediately, and sera were obtained after centrifugation at 1000×***g*** for 20 min and stored at −80°C until use. Serum levels of hepatic function markers were determined using commercial kits (Human Assay Kits, Wiesbaden, Germany). Simultaneously, liver tissues were homogenized in ice-cold phosphate-buffered saline (PBS) (pH 7.0). Aliquots of tissue homogenates were used to measure malondialdehyde (MDA) levels, antioxidant enzyme activity [i.e. catalase (CAT), superoxide dismutase (SOD), glutathione peroxidase (GPx), and glutathione reductase (GR)] and glutathione (GSH) content following the instructions supplied with relevant assay kits (Bio-Diagnostic, Giza, Egypt). For NO, the Biodiagnostic Nitrite Assay Kit was used which provides an accurate and convenient method for measurement of endogenous nitrite concentration as indicator of NO production. In addition, enzyme-linked immunosorbent assay (ELISA) kits were used to measure the hepatic levels of TNF-α and IL-6 (BioSource International Inc., Camarillo, CA, U.S.A.). The concentration of protein was determined using the method of Bradford [26], and crystalline bovine serum albumin was used as a standard.

### Electron microscopy of the rat liver

Small pieces of rat liver were immediately fixed in 3% glutaraldehyde in sodium phosphate buffer (200 mM, pH 7.2) for 3 h at 4°C, followed by postfixation in 1% osmium tetroxide (cold) for 1 h. The tissue samples were dehydrated in graded ethanol solutions and embedded in Araldite. Next, blocks were trimmed and sectioned with a Leica EM UC6 ultramicrotome. Ultrathin sections (80–100 nm) were mounted on 200-mesh Cu grids, double stained with 4% uranyl acetate (15 min) and 1% lead citrate (2 min). Stained sections were examined under a transmission electron microscope (Jeol JEM 1011) at 80 kV.

### Cell culture

Low-passage HepG2 cells were cultured in Dulbecco’s modified Eagle’s minimum essential medium (DMEM, Gibco), supplemented with 10% heat-inactivated fetal bovine serum (FBS), 50 IU/ml penicillin/streptomycin (Lonza Co., Belgium) at 37°C in a 5% CO_2_ atmosphere. Cells were routinely subcultured and washed with PBS. Cells were treated at 70–80% confluence.

### Cell viability assay (MTT assay)

HepG2 cells were cultured as described above. A total of 5–10 × 10^4^ cells/well were seeded in a 96-well plate and allowed to attach and grow to optimal densities. Cells were treated with increasing concentrations of MTX and/or Nar. Controls were performed in which cells were treated with 0.1–1% DMSO. After 48 h of incubation, the cells were washed twice with PBS. Then, 10 μl of 12 mM MTT (3-(4,5-dimethylthiazol-2-yl)-2,5-diphenyltetrazolium bromide) was added to each well prior to further incubation at 37°C for 4 h. The reaction was stopped after adding 100 μl of DMSO to each well. The experiment was performed in triplicate in parallel for each concentration. The mixture was shaken on a microvibrator for 5 min, and the absorbance was read at 570 nm (*A*) using a microplate ELISA reader. The viability percentage (V %) was calculated using the following equation: $\text{V}\;\%=[(A_{\text{treated}}-A_{\text{blank}})/A_{\text{control}}-A_{\text{blank}})]\times 100$. A semilogarithmic plot of the cell viability percentage of HepG2 cells versus increasing concentrations of MTX and/or Nar was employed to calculate the absolute IC_50_ (GI_50_) which is the concentration corresponding to 50% inhibition of the viability of HepG2 cells.

### *In vitro* morphological analysis

HepG2 cells were subjected to the absolute IC_50_ of MTX and/or Nar for 48 h at 37°C in 5% CO_2_. Experiments included controls exposed to 0.1–1% DMSO. After the incubation period, the cells were washed with PBS, fixed with 10% buffered formalin, examined, and photographed using an inverted phase contrast microscope (Optika, Italy) at ×400 magnification.

### Western blotting analysis

Treated and untreated cancer cells were lysed in a 1% NP-40-containing buffer supplemented with a 1× cocktail of protease inhibitors (Roche, Germany) and phosphatase inhibitors (Sigma–Aldrich, U.S.A.) at 4°C for 30 min. Lysates were centrifuged at 10000×***g*** at 4°C for 15 min, and the supernatants were collected. Protein concentrations were determined using the BCA Protein Assay Kit (Pierce, Rockford, IL). Samples were mixed at a ratio of 1:2 in Laemmli buffer and denatured by heating at 98°C for 5 min. A total of 40 μg of total protein was separated on 10% Tris-SDS/PAGE gels at 100 V for 1 h. For Western blotting, the separated proteins were electrophoretically blotted on to a prewetted nitrocellulose membrane (Bio-Rad Laboratories, U.S.A.) in Tris-buffered saline with Tween-20 (TBST; 20 mM Tris/HCl, pH 7.6, 137 mM NaCl) at 70 V for 2 h. Membranes were blocked for 30 min in blocking buffer (5% nonfat dry milk) followed by washing in TBST. Blotted proteins were probed with specific primary antibodies (Santa Cruz Biotechnology, U.S.A.) overnight at 4°C prior to incubation for 1 h in the appropriate horseradish peroxidase-conjugated secondary antibody (Santa Cruz Biotechnology, U.S.A.). β-Actin (Sigma–Aldrich, U.S.A.) was used as a loading control. An ECL kit was used for detection according to the manufacturer’s instructions (Amersham, U.K.). Densitometry quantification of protein bands was carried out using Quantity One software (Bio-Rad Laboratories, U.S.A.).

### Statistical analysis

Data were expressed as the mean ± standard error (SE), and the differences among groups were evaluated by one-way analysis of variance (ANOVA) followed by Tukey’s test. In all cases, differences were considered statistically significant at *P*<0.05. All statistical analyses were performed using GraphPad Prism 5.0 (GraphPad Prism Software Inc., San Diego, CA, U.S.A.).

## Results

### Nar alleviates MTX-induced liver dysfunction in rats

The levels of serum ALT, AST, ALP, and total bilirubin in the MTX group were significantly increased compared with those in the control group (Table 1). However, in the MTX + Nar groups, the abovementioned parameters significantly decreased compared with those of the MTX group. Indeed, the highest dose of Nar (80 mg/kg) markedly normalized the excessive levels of serum ALT, ALP, and bilirubin induced by MTX.

**Table 1:** Effect of Nar on liver function enzymes and bilirubin level in MTX-treated rats

Groups	ALT (U/l)	AST (U/l)	ALP (U/l)	Bilirubin (mg/dl)
**Control**	38.8^1^ ± 3.8	53.0^2^ ± 7.1	43.7^1^ ± 7.2	0.3^1^ ± 0.0
**MTX**	77.3^3^ ± 9.8	157.2^3^ ± 15.8	112.0^3^ ± 8.5	0.8^3^ ± 0.1
**MTX**+**Nar20**	54.0^4^ ± 5.7	100.6^4^ ± 13.3	80.4^4^ ± 3.5	0.5^4^ ± 0.1
**MTX**+**Nar40**	51.2^4^ ± 4.8	98.7^4^ ± 7.9	74.3^4^ ± 5.1	0.6^4^ ± 0.1
**MTX**+**Nar80**	36.0^1^± 3.5	70.8^1^ ± 3.7	50.0^1^ ± 2.5	0.37^1^ ± 0.0

Data are presented as mean ± SE (*n*=6).

Mean values with different superscript numbers in the same column are significantly different at *P*<0.05.

### Nar ameliorates MTX-induced hepatic oxidative stress in rats

The results of the oxidant status of the liver tissue are presented in Table 2. We found a significant reduction in SOD, CAT, GPx, GR, and GSH in rats treated with MTX. Nar administration inhibited the decrease in enzymatic activities and GSH levels in a dose-dependent manner compared with the MTX administration. Interestingly, Nar at 80 mg/kg restored CAT activity to normal levels. It was also evident that treatment with MTX significantly increased MDA and NO levels when compared with the ‘no treatment’ control. However, MDA and NO levels were much lower in the MTX+Nar group than in the MTX group.

**Table 2:** Effect of Nar on oxidative stress parameters in MTX-treated rats

Groups	MDA nmol/g tissue	GSH μmol/g tissue	CAT U/mg protein	SOD U/mg protein	GPx mU/mg protein	GR U/mg protein	Nitrite (NO) μmol/g tissue
**Control**	8.4^1^ ± 0.1	43.7^2^ ± 1.4	44.0^2^ ± 1.1	64.0^2^ ± 1.0	68.8^2^ ± 1.3	56.2^2^ ± 0.9	6.4^1^ ± 0.1
**MTX**	36.4^2^ ± 1.0	19.0^1^ ± 1.5	22.8^3^ ± 1.5	30.2^1^ ± 0.9	32.0^1^ ± 1.6	25.8^1^ ± 1.7	21.2^2^ ± 0.8
**MTX**+**Nar20**	26.3^4^ ± 1.5	26.0^3^ ± 1.2	35.2^4^ ± 1.1	42.3^3^ ± 1.2	44.0^3^ ± 1.1	35.7^3^ ± 1.7	14.7^4^ ± 0.7
**MTX**+**Nar40**	24.7^4^ ± 1.5	25.0^3^ ± 1.5	33.3^4^ ± 1.3	47.2^3^ ± 1.0	46.0^3^ ± 1.8	39.0^3^ ± 1.3	12.5^4^ ± 0.6
**MTX**+**Nar80**	19.8^3^ ± 1.3	31.5^4^ ± 1.0	40.5^2^ ± 1.3	58.7^4^ ± 1.1	53.3^4^ ± 1.7	49.0^4^ ± 1.5	9.1^3^ ± 0.7

Data are presented as mean ± SE (*n*=6).

Mean values with different superscript numbers in the same column are significantly different at *P*<0.05.

### Nar ameliorates MTX-induced proinflammatory cytokines in the rat liver

As shown in Table 3, the MTX-treated rats exhibited a significant increase in the levels of hepatic TNF-α and IL-6 compared with the control rats. In contrast, Nar treatments induced a significant decrease in the levels of these cytokines compared with MTX treatment. In particular, the highest tested dose of Nar was more effective than the two lower doses in reducing the elevated levels of IL-6 and TNF-α following MTX treatment.

**Table 3:** Effect of Nar on proinflammatory cytokines in MTX-treated rats

Groups	TNF-α (pg/ml)	IL-6 (pg/ml)
Control	45.2^1^ ± 1.1	63.3^1^ ± 0.6
MTX	146.2^2^ ± 1.3	166.2^2^ ± 0.8
MTX+Nar20	75.7^3^ ± 1.2	86.7^3^ ± 1.2
MTX+Nar40	78.8^3^ ± 0.6	83.2^3^ ± 1.4
MTX+Nar80	57.8^4^ ± 1.2	74.7^4^ ± 0.8

Data are presented as mean ± SE (*n*=6).

Mean values with different superscript numbers in the same column are significantly different at *P*<0.05.

### Nar improves MTX-induced hepatocyte ultrastructural changes in rats

**Figure 1:**
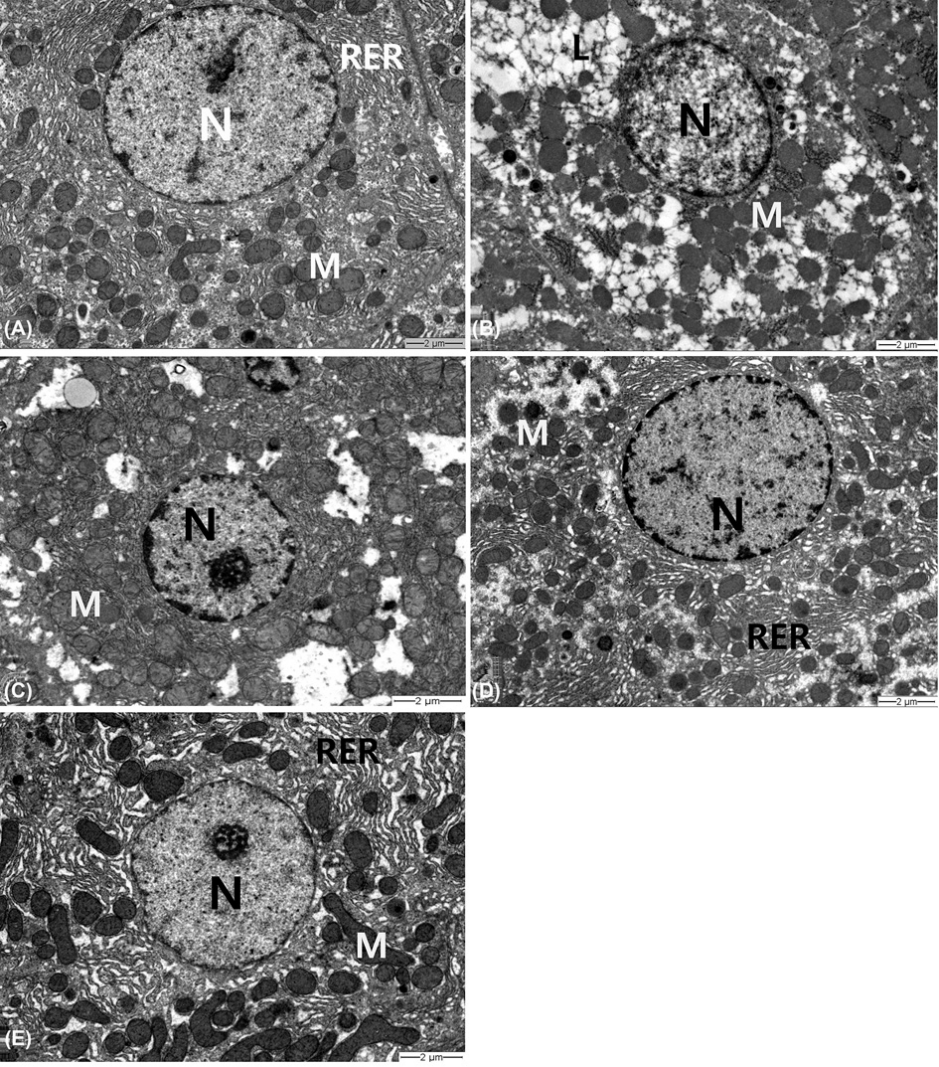
Representative transmission electron micrographs of rat hepatocytes (**A**) Control, (**B**) MTX, (**C**) MTX+Nar20, (**D**) MTX+Nar40, and (**E**) MTX+Nar80. Abbreviations: L, lipid droplet; M, mitochondria; N, nucleus; RER, rough endoplasmic reticulum

Electron microscopic examination of liver sections of control rats displayed normal architecture (Figure 1A). The cytoplasm of hepatocytes contained abundant mitochondria with well-developed cristae. The rough endoplasmic reticulum (RER) consisted of closely packed parallel and flattened cisternae. The nuclei appeared spherical with a small amount of heterochromatin at the peripheral regions and a large central amount of euchromatin. In the MTX group, RER cisternae of hepatocytes were fragmented, and mitochondria were reduced and had ill-defined cristae, karyolysis was prominent, normal structure was almost absent, and extensive fat droplets could be seen (Figure 1B). Treatment with Nar rescued the morphology of the hepatocytes and restored the cytoplasmic organelles, more strongly with increasing dosage. Although slight degeneration was observed in the cytoplasm of liver cells treated with low and medium doses of Nar, less fragmented RER was observed, and mitochondria were well-protected (Figure 1C,D). The high dose of Nar significantly prevented ultrastructural damage to the hepatocytes (Figure 1E).

### Nar enhances the cytotoxicity of MTX against HepG2 cells

**Figure 2:**
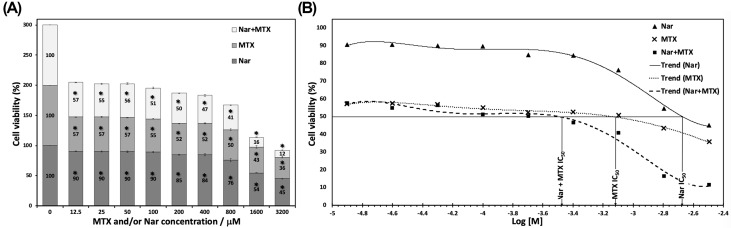
Nar enhanced the cytotoxicity of MTX against HepG2 cells Cells were treated with increasing concentrations of Nar and/or MTX (12.5 μM–3.2 mM) for 48 h. Controls were treated with DMSO only. (**A**) The effects of MTX and/or Nar on the proliferation of HepG2 cells are expressed as a percentage of cell viability. (**B**) A semilogarithmic plot of the cell viability percentage of HepG2 cells over the 48 h exposure to increasing concentrations of MTX and/or Nar. IC_50_, absolute IC_50_ is the concentration that corresponds to 50% inhibition of the viability of HepG2 cells. Data from at least three independent experiments performed in at least triplicate are presented as the means ± SEM, *P<0.01 vs. control group.

To investigate the effect of MTX and/or Nar on the growth inhibition of HepG2 cells, an MTT assay was employed. Compared with the respective untreated control cells, cells treated with increasing concentrations of MTX and/or Nar (12.5 μM–3.2 mM) for 48 h exhibited decreased cell viability. As indicated in Figure 2A, compared with control cells, the significant decline in the viability of MTX- and/or Nar-treated HepG2 cells started after the treatment with the lowest doses of MTX and/or Nar (*P*<0.01) and was significantly dose-dependent. MTX treatments exhibited more efficient inhibition (absolute IC_50_ = 0.8 mM) viability than Nar treatments (absolute IC_50_ = 2 mM). Noticeably, the inhibition of the viability of HepG2 cells was significantly improved after cotreatment with MTX and Nar (absolute IC_50_ = 0.3 mM for both) (Figure 2B).

### MTX and/or Nar induce morphological alterations in HepG2 cells

**Figure 3:**
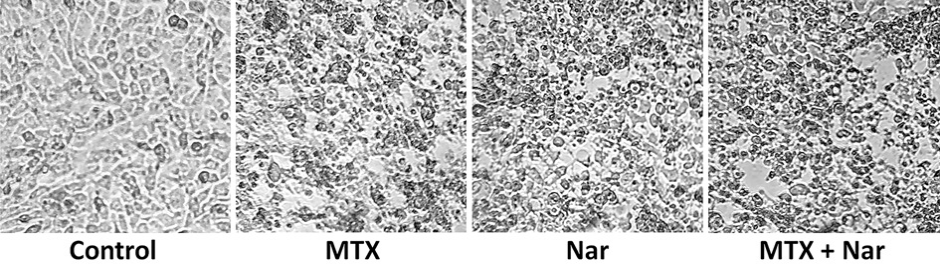
MTX- and/or Nar-induced morphological alterations in HepG2 cells Cells were plated in 12-well plates and treated with the absolute IC_50_ of MTX and/or Nar for 48 h. The photographs were taken from culture plates using a phase contrast microscope at ×400 magnification. Control cells were treated with DMSO only. Representative pictures are shown from three independent experiments.

We evaluated the effect of MTX and/or Nar treatment on HepG2 cell morphology after a 48-h treatment period. The untreated cells showed a high confluency rate and normal monolayer formation compared with treated cells. The morphological changes of treated cells are shown in Figure 3. Morphological alterations and decreases in HepG2 cell dimensions and density were observed in treated cells when compared with control cells. Treated cells exhibited a disturbed monolayer, swelling and rounded morphology with condensed chromatin; cells became shrunken, rounded in shape, showing blebbing and an increased number of floating cells, while untreated cells had an intact appearance.

### Nar potentiates the MTX effect on the Bax/Bcl-2 ratio in HepG2 cells

**Figure 4:**
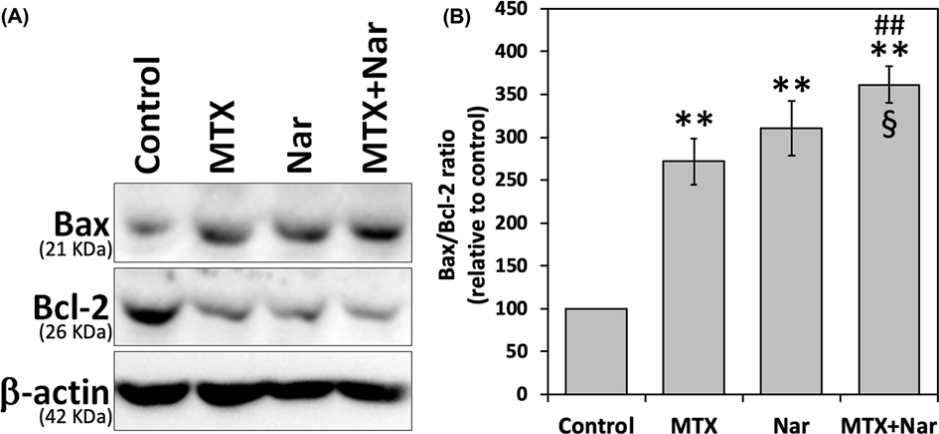
Bax and Bcl-2 expression levels and Bax/Bcl-2 ratio in MTX and/or Nar-treated HepG2 cells Cells were plated in six-well plates and treated with the absolute IC_50_ of MTX and/or Nar for 48 h. (**A**) Expression levels of Bax and Bcl-2 in HepG2 cells. (**B**) Bax/Bcl-2 ratio in HepG2 cells. Data from at least three independent experiments are presented as the means ± SE. Significant difference with control (**P<0.01). Significant difference with the MTX group (^##^*P*<0.01). Significant difference with the Nar group (^§^*P*<0.05).

To further investigate the effect of MTX and/or Nar on HepG2 cells, the expression levels of the proapoptotic protein Bax and the antiapoptotic protein Bcl-2, which are involved in proliferation, were evaluated. HepG2 cells were treated with the absolute IC_50_ of Nar and/or MTX for 48 h. The general profile of cells after different treatments, increased Bax/Bcl-2 ratio, was similar when compared with control untreated cells. Noticeably, the Bax/Bcl-2 ratio was significantly increased after cotreatment with MTX and Nar (Figure 4).

## Discussion

In the present study, MTX-induced hepatotoxicity was manifested by a significant increase in the levels of serum ALT, AST, and ALP enzymes. These enzymes are specific indicators of increased membrane permeability and/or necrotic damage in hepatocytes [27]. In addition, MTX induced a concomitant increase in total bilirubin levels, which is also a clear marker of hepatic dysfunction [28]. Liver injury was also confirmed by ultrastructural deformations in hepatocytes in the form of lipid infiltration, pyknotic or karyolysed nuclei, mitochondrial cristolysis, and RER fragmentation. These changes are consistent with previous studies [29,30]. Treatment of the MTX-administered rats with Nar significantly ameliorated all circulating liver function markers. In addition, Nar markedly protected cellular membranes and organelles with increasing doses, to the point that normal hepatocyte ultrastructure returned. Similar findings have been reported by Lv et al. [31], who showed that Nar supplementation led to the resumption of elevated serum hepatic enzyme activities in perfluorooctane sulfonate-challenged mice. Another study by Zhou et al. reported that Nar reduced alcoholic hepatic steatosis, and the inhibitory effect was dose-dependent [32].

MTX can initiate hepatocellular oxidative damage through the excessive generation of ROS, mitochondrial dysfunction, and deregulation of enzymatic or nonenzymatic antioxidant machinery [33,34]. It has been reported that cytosolic NADP^+^-dependent dehydrogenases and NADP^+^-malic enzymes are inhibited by MTX, suggesting that the drug could decrease the availability of intracellular NADPH in cells, leading to depletion of cellular GSH and making cells more vulnerable to damage by ROS [35]. ROS generation may lead to LPO and damage to cellular macromolecules, such as DNA, proteins, and lipid bilayers [36]. Under the current experimental conditions, MTX administration induced a significant increase in LPO products (i.e., MDA) and a significant decrease in endogenous antioxidants, including GSH, CAT, SOD, GPx, and GR. This increase in redox imbalance was ameliorated by Nar treatment in a dose-dependent manner, possibly by its direct ability to scavenge free radicals [37], or through the transcriptional up-regulation of antioxidant genes [38]. These results are consistent with reports of other investigators [31,39,40]. Several studies have reported that the antioxidant and anti-inflammatory effects of Nar are retained up to 400 mg/kg exposure [41–43]. In addition, Nar have a high safety margin toward cultured primary rat hepatocytes (IC_50_ = 99.6 g/ml) [44].

MTX administration has been shown to produce a significant increase in the levels of proinflammatory cytokines, such as TNF-α and IL-6, as well as NO production [4,45]. Excess NO in the presence of superoxide anions may generate peroxynitrite radicals, cytotoxic prooxidants that causes protein nitration and cellular damage [46]. In addition, excess NO depletes intracellular GSH levels, thus increasing the susceptibility to oxidative stress [47]. The results of the present study revealed that the increase in TNF-α, IL-6, and NO levels was inhibited by Nar treatment when supplemented prior to and after MTX administration. This finding is consistent with previous studies [48,49].

iNOS is not constantly present in cells and accumulating evidences point out the involvement of iNOS in the production of NO when the cell is induced or stimulated, typically by proinflammatory cytokines and/or bacterial lipopolysaccharide. Upon induction, iNOS generates significant amounts of NO (micromolar range), which lasts sometimes for hours. Constitutive NOS (cNOS), by contrast, generate and release NO mainly in resting cells and produce nanomolar amounts of NO for short periods of time [50,51]. Different experimental works have shown that cNOS activity is markedly inhibited by NO itself, whereas iNOS activity is highly resistant to inhibition by NO [52]. It is well established that proinflammatory cytokines are potent inducers of iNOS in a wide variety of cells types, with consequent production of NO [53]. The gene expression of iNOS and the subsequent mRNA translation are regulated by various stimuli, especially endogenous proinflammatory mediators such as TNF-α, IL-1β, IL-6, and IFN-γ. Hence, the expression of iNOS can only be induced by inflammatory stimuli and contribute to the large amount of NO production [50]. The mechanism for the Nar anti-inflammatory action might involve the inhibition of NF-κB and iNOS signaling pathway [31,37].

Regardless of the combined efforts of governments and researchers globally, there has been a continuous rise in the incidence rate of HCC during the last two decades [54]. Toward the second aim of the present study, HepG2 cells, as a model for HCC, were used to investigate the possible cytotoxic and antiproliferative effects of MTX and/or Nar. MTX and/or Nar provoked evident antitumor activity in HepG2 cells, as assessed by *in vitro* cytotoxicity, morphological alterations, and the Bax/Bcl-2 protein expression ratio, which is consistent with what has been reported previously *in vitro* and *in vivo* [20,23]. The reduction in the percentage of cell viability was dose-dependent following 48 h of treatment. The MTT results showed that MTX and/or Nar significantly inhibited the proliferation of HepG2 cells. Cells were more sensitive to MTX and Nar cotreatment (absolute IC_50_ = 0.3 mM for each) than either MTX (absolute IC_50_ = 0.8 mM) or Nar (absolute IC_50_ = 2 mM) treatment alone. The treatment with MTX alone resulted in a nontypical growth inhibitory action on cancer cells with two concentration areas of inhibitory action: one below 10 μM and other above 1 mM. This most probably indicates two different inhibition mechanisms and/or two different targets. In case of the combined treatment of MTX+Nar, the inhibitory effect below 10 μM may be related to the action of MTX alone, whereas the inhibition around 1 mM may be caused by the combined action of both compounds. We did not investigate the effect of concentrations below 10 μM.

The induction of apoptosis is an important pathophysiological strategy through which an ideal antitumor agent acts to arrest cancer cell growth [55]. Apoptosis is typically characterized by morphological alterations [56]. Morphological examination of treated cells revealed apoptotic HepG2 cells with obvious changes in morphology, including cell shrinkage and nuclear chromatin condensation, implying the involvement of MTX and/or Nar, as reported previously [20].

Although the antiproliferative effect of Nar on HepG2 cells has been reported [19], the molecular mechanism involved remains unclear. Here, the immunoblotting analysis showed an up-regulation of the proapoptotic Bax and down-regulation of the antiapoptotic Bcl-2 upon treatment with MTX and/or Nar. The induction of apoptosis is a protective mechanism to eliminate neoplastic cells [57]. Apoptosis may be initiated via the activation of the extrinsic or intrinsic pathways or both. Mitochondria are involved in the intrinsic apoptosis pathway. This apoptotic pathway is regulated by Bax, Bcl-2, and cytochrome *c* and eventually leads to the activation of caspase-3 and poly (ADP-ribose) polymerase (PARP) [19,58]. The activation of caspases is regulated by the fine balance of the ratios of Bax and Bcl-2 proteins [59], a mechanism that regulates the survival/apoptosis decision of a cell. The observed altered expression of Bax and Bcl-2 upon MTX and/or Nar treatment of HepG2 cells in a manner that favors the increase in the Bax/Bcl-2 ratio could underlie the observed apoptotic effect and has been reported previously [19,20]. The oligomerization of Bax on the outer mitochondrial membrane, as a result of excessive oxidative stress, leads to cytochrome *c* release and degradation of Bcl-2 [60]. Here, while exposure to either MTX or Nar increased the Bax/Bcl-2 protein expression ratio, the combination of both treatments induced a more pronounced increase in the Bax/Bcl-2 ratio. This finding implies that the cotreatment of MTX and Nar synergistically aggravated their apoptotic effect in HepG2 cells. Many studies have reported that the deregulation of apoptosis-related proteins with a mechanism of action comparable to that of Nar, which further supports its role in apoptosis induction [20,58,60,61].

Nar can have a cytoprotective or apoptotic effect. Many studies have reported the apoptotic effect of Nar in different human cancer cell lines via various mechanisms and signaling pathways. Nar induces apoptosis in HeLa and HepG2 cells through activation of caspase-3, increased Bax/Bcl-2 ratio, and up-regulation of miR-19b expression [19–21,23,62,63]; in cervical cancer SiHa cells via death receptor and mitochondrial pathways [64]; and in gastric adenocarcinoma AGS cells via autophagic cell death through down-regulation of the PI3K/Akt/mTOR pathway, up-regulation of p21, activation of Beclin 1 and LC3B, and phosphorylation MAPK [65].

## Conclusion

In summary, the results of this experiment suggest that Nar can reverse MTX-induced hepatic damage through reduction in MDA, NO, TNF-α, and IL-6 with the abrogation of cellular antioxidant defenses. Moreover, Nar potentiates the cytotoxic and antiviability effect of MTX on HepG2 cancer cells. *In vivo* experiments using tumor-bearing animal models are required to determine if MTX and/or Nar are efficacious and safe for use as anticancer drugs.

## Abbreviations

CAT: catalase
cNOS: constitutive NOS
ELISA: enzyme-linked immunosorbent assay
GPx: glutathione peroxidase
GR: glutathione reductase
GSH: glutathione
HCC: hepatocellular carcinoma
IARC: International Agency for Research on Cancer
iNOS: inducible nitric oxide synthase
ip: intraperitoneal
LPO: lipid peroxidation
MDA: malondialdehyde
MTT: 3-(4,5-dimethylthiazol-2-yl)-2,5-diphenyltetrazolium bromide
Nar: Naringin (4′,5,7-trihydroxyflavanone-7-rhamnoglucoside)
NO: nitric oxide
NOS: nitric oxide synthase
PBS: phosphate-buffered saline
RER: rough endoplasmic reticulum
ROS: reactive oxygen species
SOD: superoxide dismutase
TBST: Tris-buffered saline with Tween-20
TNF-α: tumor necrosis factor-α
